# Prevalence of cognitive impairments and strengths in the early course of psychosis and depression

**DOI:** 10.1017/S0033291723001770

**Published:** 2023-10

**Authors:** Alexandra Stainton, Katharine Chisholm, Siân Lowri Griffiths, Lana Kambeitz-Ilankovic, Julian Wenzel, Carolina Bonivento, Paolo Brambilla, Mariam Iqbal, Theresa K. Lichtenstein, Marlene Rosen, Linda A. Antonucci, Eleonora Maggioni, Joseph Kambeitz, Stefan Borgwardt, Anita Riecher-Rössler, Christina Andreou, André Schmidt, Frauke Schultze-Lutter, Eva Meisenzahl, Stephan Ruhrmann, Raimo K. R. Salokangas, Christos Pantelis, Rebekka Lencer, Georg Romer, Alessandro Bertolino, Rachel Upthegrove, Nikolaos Koutsouleris, Kelly Allott, Stephen J. Wood, PRONIA Consortium, Nikolaos Koutsouleris, Dominic B. Dwyer, Lana Kambeitz-Ilankovic, Anne Ruef, Alkomiet Hasan, Claudius Hoff, Ifrah Khanyaree, Aylin Melo, Susanna Muckenhuber-Sternbauer, Yanis Köhler, Ömer Öztürk, Nora Penzel, David Popovic, Adrian Rangnick, Sebastian von Saldern, Rachele Sanfelici, Moritz Spangemacher, Ana Tupac, Maria Fernanda Urquijo, Johanna Weiske, Antonia Wosgien, Camilla Krämer, Shalaila S. Haas, Rebekka Lencer, Inga Meyhoefer, Marian Surmann, Udo Dannlowski, Olga Bienek, Georg Romer, Marlene Rosen, Theresa Lichtenstein, Stephan Ruhrmann, Joseph Kambeitz, Karsten Blume, Dominika Julkowski, Nathalie Kaden, Ruth Milz, Alexandra Nikolaides, Mauro Silke Vent, Martina Wassen, Christos Pantelis, Stefan Borgwardt, Christina Andreou, André Schmidt, Anita Riecher-Rössler, Laura Egloff, Fabienne Harrisberger, Ulrike Heitz, Claudia Lenz, Letizia Leanza, Amatya Mackintosh, Renata Smieskova, Erich Studerus, Anna Walter, Sonja Widmayer, Alexandra Korda, Rachel Upthegrove, Chris Day, Sian Lowri Griffiths, Mariam Iqbal, Mirabel Pelton, Pavan Mallikarjun, Alexandra Stainton, Ashleigh Lin, Paris Lalousis, Raimo K. R. Salokangas, Alexander Denissoff, Anu Ellilä, Tiina From, Markus Heinimaa, Tuula Ilonen, Päivi Jalo, Heikki Laurikainen, Antti Luutonen, Akseli Mäkela, Janina Paju, Henri Pesonen, Reetta-Liina Säilä, Anna Toivonen, Otto Turtonen, Frauke Schultze-Lutter, Eva Meisenzahl, Sonja Botterweck, Norman Kluthausen, Gerald Antoch, Julian Caspers, Hans-Jörg Wittsack, Ana Beatriz Solana, Manuela Abraham, Timo Schirmer, Alessandro Bertolino, Linda A. Antonucci, Giulio Pergola, Ileana Andriola, Barbara Gelao, Paolo Brambilla, Carlo Altamura, Marika Belleri, Francesca Bottinelli, Adele Ferro, Marta Re, Emiliano Monzani, Maurizio Sberna, Armando D'Agostino, Lorenzo Del Fabro, Giampaolo Perna, Maria Nobile, Alessandra Alciati, Matteo Balestrieri, Carolina Bonivento, Giuseppe Cabras, Franco Fabbro, Marco Garzitto, Sara Piccin

**Affiliations:** 1 Orygen, Melbourne, Victoria, Australia; 2 Centre for Youth Mental Health, University of Melbourne, Melbourne, Victoria, Australia; 3 School of Psychology, Aston University, Birmingham, UK; 4 Institute for Mental Health and Centre for Human Brain Health, University of Birmingham, Birmingham, UK; 5 Department of Psychiatry and Psychotherapy, Faculty of Medicine and University Hospital of Cologne, Cologne, Germany; 6 Faculty of Psychology and Educational Sciences, Department of Psychology, Ludwig-Maximilian University, Munich, Germany; 7 Scientific Institute, IRCCS E. Medea, Pasian di Prato, Udine, Italy; 8 Department of Neurosciences and Mental Health, Fondazione IRCCS Ca’ Granda Ospedale Maggiore Policlinico, University of Milan, Milan, Italy; 9 Department of Pathophysiology and Transplantation, University of Milan, Milan, Italy; 10 Department of Psychology, Woodbourne Priory Hospital, Birmingham, UK; 11 Department of Translational Biomedicine and Neuroscience (DiBraiN), University of Bari “Aldo Moro”, Bari, Italy; 12 Department of Electronics, Information and Bioengineering, Politecnico di Milano, Milan, Italy; 13 Department of Psychiatry and Psychotherapy, University of Lübeck, Lübeck, Germany; 14 Department of Psychiatry, Psychiatric University Hospital, University of Basel, Basel, Switzerland; 15 Medical Faculty, University of Basel, Basel, Switzerland; 16 Department of Psychiatry and Psychotherapy, Medical Faculty, Heinrich-Heine University, Düsseldorf, Germany; 17 Department of Psychology, Faculty of Psychology, Airlangga University, Surabaya, Indonesia; 18 University Hospital of Child and Adolescent Psychiatry and Psychotherapy, University of Bern, Bern, Switzerland; 19 Department of Psychiatry, University of Turku, Turku, Finland; 20 Melbourne Neuropsychiatry Centre, University of Melbourne and Melbourne Health, Melbourne, VIC, Australia; 21 Institute for Translational Psychiatry, University of Münster, Münster, Germany; 22 Department of Child Adolescent Psychiatry and Psychotherapy, University of Münster, Münster, Germany; 23 Birmingham Early Intervention Service, Birmingham Women's and Children NHS Foundation Trust, Birmingham, UK; 24 Department of Psychiatry and Psychotherapy, Ludwig-Maximilian-University, Munich, Germany; 25 Max-Planck Institute of Psychiatry, Munich, Germany; 26 Institute of Psychiatry, Psychology and Neuroscience, King's College London, London, UK; 27 School of Psychology, University of Birmingham, Edgbaston, UK

**Keywords:** Mental health, psychosis, working memory, processing speed, verbal learning

## Abstract

**Background:**

Studies investigating cognitive impairments in psychosis and depression have typically compared the average performance of the clinical group against healthy controls (HC), and do not report on the actual *prevalence* of cognitive impairments or strengths within these clinical groups. This information is essential so that clinical services can provide adequate resources to supporting cognitive functioning. Thus, we investigated this prevalence in individuals in the early course of psychosis or depression.

**Methods:**

A comprehensive cognitive test battery comprising 12 tests was completed by 1286 individuals aged 15–41 (mean age 25.07, s.d. 5.88) from the PRONIA study at baseline: HC (*N* = 454), clinical high risk for psychosis (CHR; *N* = 270), recent-onset depression (ROD; *N* = 267), and recent-onset psychosis (ROP; *N* = 295). Z-scores were calculated to estimate the prevalence of moderate or severe deficits or strengths (>2 s.d. or 1–2 s.d. below or above HC, respectively) for each cognitive test.

**Results:**

Impairment in at least two cognitive tests was as follows: ROP (88.3% moderately, 45.1% severely impaired), CHR (71.2% moderately, 22.4% severely impaired), ROD (61.6% moderately, 16.2% severely impaired). Across clinical groups, impairments were most prevalent in tests of working memory, processing speed, and verbal learning. Above average performance (>1 s.d.) in at least two tests was present for 40.5% ROD, 36.1% CHR, 16.1% ROP, and was >2 SDs in 1.8% ROD, 1.4% CHR, and 0% ROP.

**Conclusions:**

These findings suggest that interventions should be tailored to the individual, with working memory, processing speed, and verbal learning likely to be important transdiagnostic targets.

## Introduction

Cognitive impairments are a prominent feature of early-stage mental illnesses, particularly in full-threshold psychotic disorder (Fioravanti, Bianchi, & Cinti, 2012; Mesholam-Gately, Giuliano, Goff, Faraone, & Seidman, 2009), clinical high-risk (CHR) for psychosis (Catalan et al., 2021; Fusar-Poli et al., 2012; Pukrop et al., 2006), and major depressive disorder (MDD; Ahern & Semkovska, 2017; Goodall et al., 2018). Such impairments can significantly impact an individual's everyday functioning and long-term outcomes. Cognition has been found to be predictive of progression through the psychosis spectrum (Koutsouleris et al., 2011). Further, in a transdiagnostic sample of young people with mental illness, baseline cognition was the strongest predictor of two-year functional outcomes (Lee et al., 2013). While the literature to date has clearly demonstrated a meaningful link between mental health difficulties and cognitive impairments, studies have largely taken a group-level approach to analysis. Such studies have shown that individuals with mental illnesses, on average, perform more poorly than healthy controls (HC) in multiple cognitive domains (East-Richard, R-Mercier, Nadeau, & Cellard, 2020). However, statistically significant differences at a group level cannot necessarily equal a clinically meaningful impairment (Abramovitch & Schweiger, 2015; Abramovitch, Short, & Schweiger, 2021; Michel, Ruhrmann, Schimmelmann, Klosterkötter, & Schultze-Lutter, 2014). At present there is no universally agreed definition of a *clinically meaningful* cognitive impairment (Abramovitch et al., 2021). However, by examining the amount to which individual performance deviates from the average (standard deviation difference), we can obtain a clearer picture of how common moderate or severe cognitive difficulties may be within early intervention services, and who may require further cognitive assessment or treatment.

Clinical guidelines for early psychosis (e.g. Early Psychosis Guidelines Working Group, 2016; NICE, 2014) and depression (e.g. Malhi et al., 2021; National Institute for Clinical Excellence, 2009) recommend an assessment of cognition and remediation therapies where cognition may be impairing functional recovery. Thus, it is important to estimate the prevalence of people presenting to services with impaired cognition who may need to access those additional services. This would allow for more adequate resourcing of clinical services and could inform relevant training for clinicians. Furthermore, because there may be a variation in the cognitive strengths or impairments in people presenting to services, it is important to screen cognition at service entry (Bryce & Allott, 2019; Bryce, Bowden, Wood, & Allott, 2021), and consider the cognitive profile of individuals who are in the early course of mental illness. Cognitive impairments are often present long before the onset of clinical symptoms (e.g. Mollon, David, Zammit, Lewis, & Reichenberg, 2018), and may also show further decline following the first-episode of psychosis (Fett et al., 2020; Flaaten et al., 2022), or with multiple depressive episodes (Allott, Fisher, Amminger, Goodall, & Hetrick, 2016; Semkovska et al., 2019). Thus, understanding the profile of cognitive performance in the early course of illness will allow clinicians to intervene early to preserve intact cognitive skills or prevent further decline (Pantelis, Wannan, Bartholomeusz, Allott, & McGorry, 2015), providing the best possible chance of functional recovery.

The idea that a subgroup of individuals with schizophrenia may be ‘neuropsychologically normal’ has been previously discussed (Keefe, 2008; Wilk et al., 2005), and some more recent studies have used data-driven approaches, which identified clusters of relatively spared *v.* impaired cognitive performance in early psychosis (Gould et al., 2014; Uren, Cotton, Killackey, Saling, & Allott, 2017; Wenzel et al., 2021). Few studies have examined the *prevalence* of cognitive impairments in this population. Two previous reports estimated that 70–80% of individuals with chronic schizophrenia demonstrate cognitive impairments (Allen, Goldstein, & Warnick, 2003; Palmer et al., 1997). Less is known about the prevalence of cognitive impairments in the early stages of the illness. In a sample of Ugandan inpatients with first-episode psychosis, 62% presented with a cognitive impairment, defined as mean scores of >2 SDs below HC in one domain, or of >1 SDs below HC in two or more domains (Mwesiga et al., 2022). Furthermore, to the best of our knowledge there are no previous studies which have investigated the prevalence of cognitive impairments in CHR.

The prevalence of cognitive impairments in MDD has been investigated in a few studies. Gualtieri and Morgan (2008) observed that up to 32% of their treated MDD sample had an impairment of 1–2 SDs relative to HC in at least one cognitive domain, which was >2 SDs in up to 18%. When cognitive impairment is defined as being >1 s.d. below HC in at least two cognitive tests, Douglas et al. (2018) observed a prevalence of 78.6% in their outpatient MDD sample, which was >2 SDs below HC in 14.3% of the sample. They also observed that their inpatient depression group had higher rates of impairment, at 91.4% (>1 s.d. in two tests) and 32.8% (>2 SDs in two tests). Tran, Milanovic, Holshausen, and Bowie (2021) also investigated the prevalence of impairment relative to the individuals' estimated premorbid performance in a community sample of individuals with MDD. They identified that 62.2% of the sample were performing at least 1 s.d. below their estimated premorbid performance. Finally, some studies have calculated the prevalence of a composite cognitive impairment (the average of cognitive test scores). The prevalence of composite impairment in outpatient MDD samples has ranged from 11.8% (Douglas et al., 2018) to 25.2% (Tran et al., 2021) at a level of >1 s.d. relative to HC, and has been observed in 1.5% of an outpatient MDD sample at >2 SDs relative to HC (Douglas et al., 2018). Thus, while rates of impairment appear high in adults with established MDD, less is known about the prevalence of cognitive impairment in younger recent-onset samples.

Beside understanding the prevalence of cognitive impairments during the early stages of serious mental illnesses, it is equally important to consider the prevalence of cognitive *strengths*. Current approaches to treatment of cognition often focus on the remediation of deficits. However, reinforcing and building on strengths could be useful as an adjunct to such remediation (Allott et al., 2020), to prevent further deterioration (Pantelis et al., 2015), and to leverage important psychological factors for the treatment process such as self-esteem and motivation (Allott et al., 2020). By understanding the prevalence of cognitive strengths, we can provide clinicians and services with a more comprehensive picture of cognition during the first-episode, which could be used to inform service delivery and training. To our knowledge, no study has yet examined the prevalence of cognitive strengths in mental illness.

In summary, the studies conducted to date have demonstrated that there is a significant association between serious mental disorders and cognitive impairment at the group level. Further, individuals with psychosis or depression may exhibit poorer performance on cognitive tasks than they would have if they had never developed the illness (Keefe, Eesley, & Poe, 2005; Tran et al., 2021). However, few studies have reported the prevalence of cognitive impairments in the earliest stages of mental illness, and no studies have reported the prevalence of cognitive strengths. We aimed to report the prevalence of clinically meaningful levels of cognitive strengths or impairments in a multi-diagnostic sample of individuals diagnosed with recent-onset psychosis (ROP) or depression, or identified at clinical high-risk for psychosis. We categorised cognitive test performance at various levels ranging from >2 SDs above average to >2 SDs below average. We defined impairments and strengths as both moderate (1–2 SDs) and extreme (>2 SDs) in two or more tests, as well as report a composite cognitive score across these levels. This definition allows for comparison with previous literature (e.g. Douglas et al., 2018; Tran et al., 2021), and, in the case of impairment, highlights that which might require clinical attention.

## Methods

### Participants

This sample comprised 1286 individuals aged 15–41 who were recruited into the multi-site ‘Personalised Prognostic Tools for Early Psychosis Management’ (PRONIA; https://www.pronia.eu) study and completed a cognitive test battery at baseline. Although the maximum age for inclusion was 40, one participant in the HC group was assessed on the date of their 41^st^ birthday and retained in analysis. Full details on the methods and recruitment of the PRONIA study are provided in Koutsouleris et al. (2018). In summary, participants were recruited from ten international sites (Germany: Munich, Cologne, Münster, and Düsseldorf; UK: Birmingham; Italy: Udine, Bari, and Milan; Finland: Turku; Switzerland: Basel) if they met criteria for ROP, recent-onset depression (ROD), or clinical high risk for psychosis (CHR), or for HC with no personal or family history of mental illness. Inclusion and exclusion criteria for each study group are summarised in online Supplementary Table S1. All participants provided their written consent (or assent for participants aged <18) after having received all study information. All sites gained approval from their respective ethics committees.

### Procedure

At baseline, participants were screened for inclusion and then completed the cognitive test battery and demographic measures including age, sex, education, and ethnicity. Cognitive tests were completed in a standardised order and in the native language of each site.

### Measures

The cognitive test battery comprised the following tests and primary outcome scores (also see online Supplementary Material, ‘Description of Cognitive Tasks’ and online Supplementary Table S2): Trail Making Test (TMT) parts A & B, total reaction time (Army Individual Test Battery, 1944); phonetic (PVF) and semantic (SVF) verbal fluency, total number of correct words (Benton, Hamsher, & Sivan, 1994); Continuous Performance Task (CPT), d-Prime Sensitivity Index (Cornblatt, Risch, Faris, Friedman, & Erlenmeyer-Kimling, 1988); Rey Auditory Verbal Learning Test (RAVLT), sum of trials 1–5 (Rey, 1964); Rey-Osterrieth Complex Figure (ROCF), total score at immediate recall (Osterrieth, 1944; Rey, 1941); Self-Ordered Pointing Task (SOPT), total errors (Petrides & Milner, 1982); Auditory Digit Span task, number of correct trials forwards (FDS) and backwards (BDS) (Wechsler, 1939); Digit-Symbol Substitution Test (DSST), total score of number correct subtracted by number of errors (Copyright free version, component of the Wechsler Adult Intelligence Scale-Revised; Wechsler, 1981); Diagnostic Analysis of Non-Verbal Accuracy (DANVA), total number of correct faces identified (Nowicki & Duke, 1994); and the vocabulary and matrix reasoning subscales of the Wechsler Abbreviated Scale of Intelligence (Wechsler, 2011). These tests were chosen to represent a comprehensive evaluation of the participant's cognitive functioning. Results were reported according to individual test scores to facilitate use of the findings for clinicians. Clinicians may use different cognitive test batteries in practice and thus, by reporting individual tests they will be able to directly check the expected prevalence of strength or impairment in a clinical population, relative to controls, on a particular test.

### Statistical analysis

Statistical analysis was completed using IBM SPSS Statistics 25 software (IBM Corp, 2017). Group differences on demographic variables were examined using one-way ANOVA for continuous variables, or chi-squared for categorical variables. Post-hoc analysis then determined the direction of any significant group effects, using Tamhane or Bonferroni adjustment. The primary outcome scores for each cognitive test were chosen, with any ‘reaction time’ or ‘error’ scores reversed by subtracting the individual raw score from the highest raw score on that test, so that higher scores always indicated better performance. Following this, z-scores based on the mean and standard deviation (s.d.) of the HC study group were calculated using the formula: (raw score – HC mean) / HC s.d.. A ‘composite’ cognitive score was computed for each individual as the average of all twelve z-scores. Missing data values were small in this dataset, particularly in eleven of the twelve cognitive tasks (0.7–5.4%). However, more data was missing on the RAVLT (12.8%), as an alternative measure was used by the Finnish site, and those Finnish data were not used in the current study. The prevalence of various levels of cognitive impairment or strength was then investigated by allocating the proportion of z-scores for each cognitive test into the following categories: >2 SDs below HC (severely impaired); 1–2 SDs below HC (moderately impaired); ‘average performance’, meaning that performance was within 1 s.d. relative HC; 1–2 SDs above HC (above average), and >2 SDs above HC (extremely high). Chi-square analysis examined whether there was a significantly different proportion of the study groups meeting each level of cognitive performance. Post-hoc comparisons (with Bonferroni correction for multiple comparisons) were conducted to examine any group differences in each level of cognitive performance for each individual test. For each test and the composite score, we also calculated the odds ratio of each clinical group demonstrating a cognitive impairment (anything >1 s.d.) relative to HC and the 95% confidence intervals of the odds ratio, using the formula proposed by Altman (1990). Next, we examined the proportion of each study group showing moderate or severe cognitive impairment, or above average performance on a given number of tests. Cognitive impairment (or strength) was reported at the level of both 1–2 and >2 SDs relative to HC in at least two tests, or in the composite score. This definition was chosen to facilitate comparison with previous research (e.g. Douglas et al., 2018; Tran et al., 2021). For each participant group, the mean number of tests impaired or above average at both levels was also calculated.

## Results

The characteristics of the sample are reported in Table 1. The four study groups significantly differed on age, sex, site, IQ estimate, years of education, and ethnicity. Post-hoc examinations of these group differences are shown in Table 1. Mean raw cognitive scores for each study group are presented in online Supplementary Table S3.

**Table 1.** Characteristics of the sample

	Whole sample (*N* = 1286)	HC (*N* = 454)	ROD (*N* = 267)	CHR (*N* = 270)	ROP (*N* = 295)	Statistic	*p* value	Post-hoc
Age								CHR<HC, *p* = 0.003* CHR<ROD, *p* = 0.001* CHR<ROP, *p* < 0.001*
Mean (s.d.)	25.07 (5.88)	25.17 (5.99)	26.64 (6.13)	23.67 (5.26)	25.67 (5.84)	*F* = 6.939	<0.001*	
Range	15–41^a^	15–41^a^	15–40	15–40	15–40			
Gender								ROP>HC (Male), *p* < 0.001* ROP>CHR (Male), *p* = 0.032*
Male %	47.5	40.6	49.2	46.1	57.8	χ^2^ = 21.771	<0.001*	
Estimated Current IQ								HC>CHR, *p* < 0.001* HC>ROD, *p* < 0.001* HC>ROP, *p* < 0.001* CHR>ROP, *p* < 0.001* ROD>ROP, *p* < 0.001*
Mean (s.d.)	105.58 (12.09)	110.00 (10.03)	105.54 (11.83)	105.46 (10.99)	98.92 (13.10)	*F* = 56.655	<0.001*	
Education Years								HC>CHR, *p* < 0.001* HC>ROD, *p* < 0.001* HC>ROP, *p* < 0.001* ROD>CHR, *p* = 0.014*
Mean (s.d.)	14.60 (3.12)	15.71 (3.09)	14.35 (3.06)	13.60 (2.55)	14.00 (3.20)	*F* = 34.339	<0.001*	
Ethnicity								
*N* (%)						χ^2^ = 43.591	<0.001*	ROD>ROP (White), *p* = 0.004*
White	1067 (83.0)	381 (83.9)	235 (88.0)	226 (83.7)	225 (76.3)			HC>ROD (Asian), *p* = 0.001*
Asian	72 (5.6)	39 (8.6)	4 (1.5)	11 (4.1)	18 (6.1)			ROP>ROD (Asian), *p* = 0.026*
Black	26 (2.0)	6 (1.3)	3 (1.1)	4 (1.5)	13 (4.4)			ROP>HC (Black), *p* = 0.041*
Mixed race	28 (2.2)	14 (3.1)	3 (1.1)	4 (1.5)	7 (2.4)			CHR>HC (Other), *p* = 0.045*
Other	62 (4.8)	10 (2.2)	16 (6.0)	16 (5.9)	20 (6.8)			ROD>HC (Other), *p* = 0.045*
Unknown	31 (2.4)	4 (0.9)	6 (2.2)	9 (3.3)	12 (4.1)			ROP>HC(Other), *p* = 0.008*

N, Number of Participants; s.d., Standard Deviation; F, one-way ANOVA; χ^2^, Chi-squared; *, Statistically significant at a level of *p*⩽0.05.

a *Note*: One participant in the HC group was assessed on the date of their 41^st^ birthday and retained in analysis.

### Prevalence of cognitive strengths and impairment

**Figure 1.**
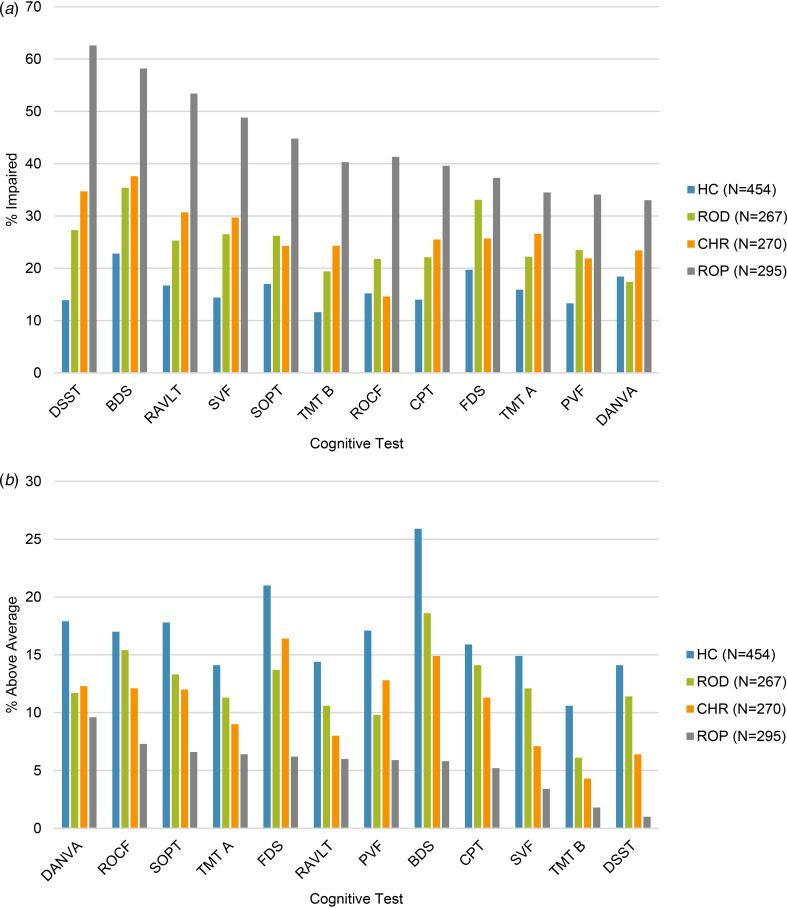
Prevalence of impairments (*a*); >1 s.d. below average and strengths (*b*); >1 s.d. above average per group in the individual cognitive tests. *Note*: Sample sizes provided are of the whole sample. Individual sample sizes for each test can be found in Table 2. Tests are presented in order from highest to lowest prevalence in the ROP group.

The percentages of each study group demonstrating each level of cognitive performance are presented in Table 2. Figures 1*a* and *b* show the prevalence of any level of impairment (e.g. anything >1 s.d. below HC) and strengths (e.g. anything >1 s.d. above HC). Though the focus of this paper was only to report the prevalence of cognitive impairments and strengths, we also examined between-group differences. Chi-square analysis showed that there was a significant difference in the proportion of participants in each study group demonstrating each level of cognitive impairment in all cognitive tests. Post-hoc analysis of significant between group differences is presented in online Supplementary Table S6. Table 2 shows the odds ratio and 95% confidence interval for that odds ratio, showing how much higher the odds of a cognitive impairment (>1 s.d. below HC) are for the clinical group compared to HC.

**Table 2.** Percentage of participants in each group meeting the criteria for each level of cognitive impairment or strengths per cognitive test

Cognitive test (*N*)	HC	ROD	CHR	ROP	χ^2^ (df)	*p* value	Odds Ratio	95% CI for Odds Ratio
**TMT A**	**(433)**	**(248)**	**(256)**	**(281)**	**χ^2^ = 49.943**	<0.001*	**ROD- 1.50**	**ROD-1.01–2.23**
>2 SDs Above	0	0.4	0	0	(12)		**CHR-1.91**	**CHR-1.31–2.79**
1–2 SDs Above	14.1	10.9	9	6.4			**ROP- 2.78**	**ROP-1.95–3.97**
No impairment	70	66.5	64.5	59.1				
1–2 SDs Below	11.1	12.1	12.9	17.4				
>2 SDs Below	4.8	10.1	13.7	17.1				
**TMT B**	**(435)**	**(247)**	**(255)**	**(280)**	**χ^2^ = 101.057**	<0.001*	**ROD- 1.67**	**ROD-1.09–2.55**
>2 SDs Above	0	0	0.4	0	(12)		**CHR- 2.22**	**CHR-1.48–3.32**
1–2 SDs Above	10.6	6.1	3.9	1.8			**ROP- 4.68**	**ROP-3.23–6.77**
No impairment	76.8	74.5	71.4	57.9				
1–2 SDs Below	8.5	8.5	11.8	19.6				
>2 SDs Below	4.1	10.9	12.5	20.7				
**Phonetic verbal fluency**	**(450)**	**(264)**	**(266)**	**(288)**	**χ^2^ = 70.502**	<0.001*	**ROD- 2**	**ROD-1.35–2.96**
>2 SDs Above	4.2	1.5	2.3	1	(12)		**CHR- 1.81**	**CHR-1.22–2.70**
1–2 SDs Above	12.9	8.3	10.5	4.9			**ROP- 3.35**	**ROP-2.33–4.83**
No impairment	69.6	66.7	65.4	60.1				
1–2 SDs Below	12	21.6	21.1	27.8				
>2 SDs Below	1.3	1.9	0.8	6.3				
**Semantic verbal fluency**	**(451)**	**(264)**	**(266)**	**(287)**	**χ^2^ = 127.906**	<0.001*	**ROD- 2.14**	**ROD-1.47–3.13**
>2 SDs Above	2.7	2.3	1.5	0.3	(12)		**CHR- 2.51**	**CHR-1.73–3.64**
1–2 SDs Above	12.2	9.8	5.6	3.1			**ROP- 5.66**	**ROP-3.98–8.03**
No impairment	70.7	61.4	63.2	47.7				
1–2 SDs Below	12	20.1	24.8	33.1				
>2 SDs Below	2.4	6.4	4.9	15.7				
**CPT d’**	**(451)**	**(263)**	**(266)**	**(288)**	**χ^2^ = 96.778**	<0.001*	**ROD- 1.74**	**ROD-1.17–2.59**
>2 SDs Above	2.2	4.6	3.4	0			**CHR- 2.12**	**CHR-1.44–3.10**
1–2 SDs Above	13.7	9.5	7.9	5.2	(12)		**ROP- 4.04**	**ROP-2.83–5.76**
No impairment	70.1	63.9	63.2	55.2				
1–2 SDs Below	12.4	17.9	24.4	30.9				
>2 SDs Below	1.6	4.2	1.1	8.7				
**RAVLT Trials 1–5**	**(389)**	**(245)**	**(238)**	**(249)**	**χ^2^ = 123.299**	<0.001*	**ROD- 1.69**	**ROD-1.14–2.50**
>2 SDs Above	0	0	0	0	(9)		**CHR- 2.21**	**CHR-1.50–3.24**
1–2 SDs Above	14.4	10.6	8	6			**ROP- 5.72**	**ROP-3.97–8.23**
No impairment	68.9	64.1	61.3	40.6				
1–2 SDs Below	12.3	15.9	20.6	25.7				
>2 SDs Below	4.4	9.4	10.1	27.7				
**ROCF Immediate memory**	**(452)**	**(266)**	**(265)**	**(288)**	**χ^2^ = 88.896**	<0.001*	**ROD- 1.55**	**ROD-1.05–2.28**
>2 SDs Above	0	0	0	0	(9)		**CHR- 1.80**	**CHR-1.23–2.64**
1–2 SDs Above	17	15.4	12.1	7.3			**ROP- 3.91**	**ROP-2.76–5.53**
No impairment	67.7	62.8	63.4	51.4				
1–2 SDs Below	11.7	14.7	12.1	20.1				
>2 SDs Below	3.5	7.1	12.5	21.2				
**SOPT total errors**	**(449)**	**(263)**	**(267)**	**(286)**	**χ^2^ = 100.049**	<0.001*	**ROD-1.75**	**ROD-1.21–2.53**
>2 SDs Above	0	0	0	0	(9)		**CHR- 1.58**	**CHR-1.09–2.29**
1–2 SDs Above	17.8	13.3	12	6.6			**ROP- 3.98**	**ROP-2.83–5.58**
No impairment	65.5	6.5	63.7	48.6				
1–2 SDs Below	12.5	19.4	14.2	21.7				
>2 SDs Below	4.5	6.8	10.1	23.1				
**Forward digit span**	**(452)**	**(263)**	**(268)**	**(292)**	**χ^2^ = 62.584**	<0.001*	**ROD- 2.02**	**ROD-1.43–2.85**
>2 SDs Above	3.3	1.9	3.7	1.4	(12)		**CHR- 1.41**	**CHR-0.99–2.02**
1–2 SDs Above	17.7	11.8	12.7	4.8			**ROP- 2.43**	**ROP-1.74–3.38**
No impairment	59.3	53.2	57.8	56.5				
1–2 SDs Below	17.5	25.9	19	26				
>2 SDs Below	2.2	7.2	6.7	11.3				
**Backward digit span**	**(451)**	**(263)**	**(269)**	**(292)**	**χ^2^ = 126.958**	<0.001*	**ROD- 1.85**	**ROD-1.32–2.58**
>2 SDs Above	0	0	0	0	(9)		**CHR- 2.03**	**CHR-1.46–2.83**
1–2 SDs Above	25.9	18.6	14.9	5.8			**ROP- 4.71**	**ROP-3.42–6.48**
No impairment	51.2	46	47.6	36				
1–2 SDs Below	22.4	35	37.2	54.1				
>2 SDs Below	0.4	0.4	0.4	4.1				
**DSST Total score**	**(453)**	**(264)**	**(265)**	**(291)**	**χ^2^ = 271.929**	<0.001*	**ROD- 2.32**	**ROD-1.59–3.39**
>2 SDs Above	2	1.9	1.5	0	(12)		**CHR- 3.29**	**CHR-2.28–4.75**
1–2 SDs Above	12.1	9.5	4.9	1			**ROP- 10.34**	**ROP-7.24–14.77**
No impairment	72	61.4	58.9	36.4				
1–2 SDs Below	13	23.5	28.7	36.1				
>2 SDs Below	0.9	3.8	6	26.5				
**DANVA Total correct**	**(453)**	**(264)**	**(269)**	**(291)**	**χ^2^ = 64.349**	<0.001*	**ROD- 0.94**	**ROD-0.63–1.40**
>2 SDs Above	0.9	1.9	0.4	0	(12)		**CHR- 1.36**	**CHR-0.94–1.97**
1–2 SDs Above	17	9.8	11.9	9.6			**ROP- 2.19**	**ROP-1.56–3.09**
No impairment	63.8	70.8	64.3	57.4				
1–2 SDs Below	13.5	12.9	16	15.5				
>2 SDs Below	4.9	4.5	7.4	17.5				
**Composite score**^a^	**(363)**	**(227)**	**(219)**	**(230)**	**χ^2^ = 138.974**	<0.001*	**ROD- 2.98**	**ROD-1.6–5.56**
>2 SDs Above	0	0	0	0	(9)		**CHR- 3.74**	**CHR-2.03–6.88**
1–2 SDs Above	1.1	0.9	0.5	0			**ROP- 12.61**	**ROP-7.24–21.97**
No impairment	94.2	86.3	84	61.7				
1–2 SDs Below	4.7	11	14.6	27.8				
>2 SDs Below	0	1.8	0.9	10.4				

χ^2^, Chi-squared examining differences in the proportion of each study group at each level of performance; *, Statistically significant at a level of *p* ⩽ 0.05; Odds Ratio, The odds of the clinical group having any impairment >1 s.d., relative to HC; CI, Confidence Interval.

a Calculated only for participants who completed all tests.

Next, we examined the proportion of each study group showing moderate or severe cognitive impairment on a given number of tests (Table 3). We observed that 88.3% of the ROP group were moderately impaired (1–2 SDs below HC) on at least two tests, and 45.1% showed severe impairment (>2 SDs below HC) on at least two tests i In the CHR group, 71.2% were moderately impaired on at least two tests, and 22.4% were severely impaired on at least two tests. In the ROD group, 61.6% were moderately impaired on at least two cognitive tests, and 16.2% were severely impaired on at least two tests.

**Table 3.** The percentage of each group demonstrating moderate or severe impairment, above average, or extremely high performance on a given number of cognitive tests

	HC	ROD	CHR	ROP	χ^2^ (df)	*p* value
**Number of tests moderately impaired (1–2 SDs Below)**					**χ^2^ = 192.472** (15)	<0.001*
0	25.6	19.8	9.1	4.3		
1	26.2	18.5	19.6	7.4		
2–3	27.5	31.3	31.5	22.2		
4–5	12.5	12.7	20.5	21.8		
6–8	6.9	9.7	15.1	25.2		
9–12	1.7	7.9	4.1	19.1		
**% Moderately Impaired on Two or More Tests**	**48.6**	**61.6**	**71.2**	**88.3**		
**Number of Tests Severely Impaired (>2 SDs Below)**					**χ^2^ = 175.494** (15)	<0.001*
0	75.8	66.1	55.7	34.3		
1	17.6	17.6	21.9	20.4		
2–3	5.8	11	16.4	23.5		
4–5	0.9	3.5	5	11.3		
6–8	0	1.7	0.5	9.1		
9–12	0	0	0.5	1.2		
**% Severely Impaired on Two or More Tests**	**6.7**	**16.2**	**22.4**	**45.1**		
**Number of Tests Above Average (1–2 SDs Above)**	**(*N* = 363)**	**(*N* = 227)**	**(*N* = 219)**	**(*N* = 230)**	**χ^2^ = 108.104** (15)	<0.001*
0	27.5	36.1	42.9	60.0		
1	20.1	23.3	21	23.9		
2–3	31.4	29.1	26.1	13.9		
4–5	15.7	8.3	7.3	1.3		
6–8	5.3	2.2	2.8	0.9		
9–12	0	0.8	0	0		
**% Above Average on Two or More Tests**	**52.3**	**40.5**	**36.1**	**16.1**		
**Number of Tests Extremely High (>2 SDs Above)**	**(*N* = 363)**	**(*N* = 227)**	**(*N* = 219)**	**(*N* = 230)**	**χ^2^ = 19.083** (6)	0.004*
0	87.3	86.3	88.6	97.0		
1	10.5	11.9	10	3		
2–3	2.2	1.8	1.4	0		
4–5	0	0	0	0		
6–8	0	0	0	0		
9–12	0	0	0	0		
**% Extremely High on Two or More Tests**	**2.2**	**1.8**	**1.4**	**0**		

χ^2^, Chi-squared; *, Statistically significant at a level of *p* ⩽ 0.05.

Regarding above average performance, 16.1% of the ROP group were above average (1–2 SDs above HC) on at least two tests, but no participant in the ROP group performed at the ‘extremely high’ level (>2 SDs above HC) on at least two tests. In the CHR group, 36.1% were above average on at least two tests, and 1.4% were extremely high on at least two tests. In the ROD group, 40.5% were above average on at least two cognitive tests, and 1.8% were extremely high on at least two cognitive tests.

Finally, the average number of impaired test performances at a moderate (1–2 SDs below) or severe (>2 SDs below) level was calculated per group (Table 4). One-way ANOVA demonstrated a significant difference between the groups in the number of tests moderately (*F*(3, 1035) = 72.810, *p* < 0.001), and severely (*F*(3, 1035) = 61.705, *p* < 0.001) impaired, as well as above average (*F*(3, 1035) = 31.708, *p* < 0.001) and extremely high (*F*(3, 1035) = 6.113, *p* < 0.001). Post-hoc examinations are presented in Table 4.

**Table 4.** Average number of tests that each study group is impaired on

	HC	ROD	CHR	ROP	*F*	*p*	Tamhane Post Hoc	CI
Number of tests moderately impaired							HC<ROD, *p* < 0.001* HC<CHR, *p* < 0.001* HC<ROP, *p* < 0.001* CHR<ROP, *p* < 0.001* ROD<ROP, *p* < 0.001*	−1.50 to −0.34 −1.79 to −0.72 −3.90 to −2.63 −2.73 to −1.29 −3.09 to −1.59
Mean (s.d.)	2.04 (2.11)	2.96 (2.84)	3.29 (2.51)	5.3 (3.22)	**72.810**	<0.001*		
Min-Max	0–10	0–12	0–12	0–12				
Number of tests severely impaired							HC<ROD, *p* = 0.001* HC<CHR, *p* < 0.001* HC<ROP, *p* < 0.001* CHR<ROP, *p* < 0.001* ROD<ROP, *p* < 0.001*	−0.65 to −0.11 −0.83 to −0.29 −2.11 to −1.27 −1.62 to −0.65 −1.79 to −0.84
Mean (s.d.)	0.35 (0.74)	0.73 (1.4)	0.91 (1.4)	2.04 (2.34)	**61.705**	<0.001*		
Min-Max	0–5	0–8	0–9	0–11				
Number of tests above average							HC>ROD, *p* = 0.022* HC>CHR, *p* < 0.001* HC>ROP, *p* < 0.001* CHR>ROP, *p* < 0.001* ROD>ROP, *p* < 0.001*	0.04–0.85 0.29–1.06 1.01–1.65 0.32–0.99 0.53–1.24
Mean (s.d.)	2.00 (1.90)	1.55 (1.75)	1.32 (1.55)	0.67 (1.06)	**31.708**	<0.001*		
Min-Max	0–8	0–10	0–7	0–6				
Number of tests extremely high							HC>ROP, *p* < 0.001* CHR>ROP, *p* = 0.003* ROD>ROP, *p* < 0.001*	0.05–0.18 0.02–0.17 0.05–0.20
Mean (s.d.)	0.15 (0.41)	0.15 (0.41)	0.13 (0.37)	0.03 (0.17)	**6.113**	<0.001*		
Min-Max	0–2	0–2	0–2	0–1				

*, Statistically significant at a level of *p* ⩽ 0.05; CI, 95% Confidence Intervals for Mean Difference.

## Discussion

This study investigated the prevalence of cognitive impairments and strengths in a sample of individuals in the early stages of psychosis or depression. While previous studies have largely examined cognitive impairment in mental illness using group-level means, we examined the proportion of individuals in each group who were demonstrating various levels of performance from extremely high to severely impaired. Overall, the findings of this study are in line with the previous literature, in that a larger proportion of the clinical groups demonstrated both moderate and severe cognitive impairment than HC. However, even within the clinical groups, a subset of participants demonstrated unimpaired cognitive performance at the individual test level, and a small proportion demonstrated above average performance.

In this sample, cognitive impairment was most prevalent in the ROP group. Previous reports have estimated that 70–80% of individuals with chronic schizophrenia (Allen et al., 2003; Palmer et al., 1997), and 62% of individuals with first-episode psychosis (Mwesiga et al., 2022), will demonstrate cognitive impairment. We observed that 88.3% of the ROP group were moderately impaired (1–2 SDs below HC) in at least two tests, and 45.1% were severely impaired (>2 SDs below HC) in at least two tests, which is a slightly higher prevalence than those previous estimates. When an average composite cognitive score was calculated, 27.8% of the ROP group were demonstrating a global cognitive impairment of 1–2 SDs below HC, and severe impairment of >2 SDs below HC in 10.4% of the sample. We also saw a much higher proportion of the present ROP group showing significant, and widespread impairment on multiple tests when compared to the other study groups. Further, our ROP group demonstrated a significantly higher average number of tests impaired at both a moderate (5.3) and severely (2.04) impaired level than all other clinical groups.

We observed that 61.6% of the ROD group were moderately (1–2 SDs below HC) impaired in at least two cognitive tests, and 16.2% were severely (>2 SDs below HC) impaired in at least two cognitive tests. When an average composite cognitive score was calculated, 12.8% of the ROD group were demonstrating a moderate cognitive impairment (1–2 SDs below HC), and severe impairment (>2 SDs below HC) was seen in 1.8% of the sample. The prevalence of impairment in at least two tests in this sample is broadly comparable to the outpatient sample of Douglas et al. (2018). They observed that 78% were moderately impaired, and 14.3% were severely impaired, according to these criteria. Furthermore, the composite impairments of 11.8% <1 s.d. and 1.5% <2 SDs in that study are comparable to the present findings. Tran et al. (2021) observed a slightly higher prevalence of global impairment <1 s.d. in 25.2% of their sample of outpatients with MDD. Their sample was slightly older than the current sample (mean age 48.6 compared to 26.6 in this ROD group), and there was no restriction to first-episode MDD. Thus, it is possible that their sample may have experienced some further cognitive decline which can be associated with repeated or prolonged depressive episodes (Allott et al., 2016; Semkovska et al., 2019).

The current CHR group demonstrated a prevalence of cognitive impairment which was comparable, but less severe, than that seen in the ROP group; 71.2% were moderately impaired in at least two tests, and 22.4% were severely impaired in at least two tests. In the composite cognitive score, 14.6% of the CHR group were demonstrating an impairment of 1–2 SDs below HC, whereas 0.9% of the sample were severe (>2 SDs below HC). To the best of our knowledge, this is the first study to investigate the prevalence of cognitive impairment in individuals at CHR for psychosis. Nevertheless, these observations align with the previous literature demonstrating that cognitive impairments can begin well before the first-episode of psychosis (Catalan et al., 2021; Fusar-Poli et al., 2012). These findings also suggest that almost three quarters of CHR populations may be experiencing moderate deficits, and almost one quarter may already be experiencing severe deficits and require cognitive interventions by the time they seek help.

Current findings can also be compared with data-driven approaches, which have broadly identified either two (Amoretti et al., 2021; Wenzel et al., 2021) or three (Uren et al., 2017) distinct clusters of cognitive performance in individuals with ROP spanning from relatively spared to severely impaired. The relatively preserved profile of cognitive performance is often associated with milder clinical symptoms and better functioning (e.g. Crouse, Moustafa, Bogaty, Hickie, & Hermens, 2018; Oomen et al., 2021; Uren et al., 2017). Similar findings have emerged for samples with mood disorders (Cotrena, Branco, Ponsoni, Shansis, & Fonseca, 2017), but there are also suggestions that in depression samples there may be separable clusters based on the *type* of domains impaired, as opposed to the severity (Hermens et al., 2011; Tickell et al., 2019). Together, previous and current findings suggest that while cognitive impairments are a core feature of mental illnesses such as psychosis and depression, there will be a subsample of individuals who are not presenting with such impairments at illness onset. This paper further extends that notion by reporting the prevalence of each group with above average cognitive performance at baseline.

When we examined the individual tests showing the largest prevalence of impairment (Table 2, Fig. 1*a*), we saw largely the same six tests in all three clinical groups; Backwards Digit Span, Forward Digit Span, Digit-Symbol Substitution Test, Rey Auditory Verbal Learning Test Trials 1–5, Self-Ordered Pointing Test Total Errors, and Semantic Verbal Fluency. These tests assess working memory, processing speed, verbal learning and memory and verbal fluency. This observation aligns with previous systematic reviews and meta-analyses, in which deficits in these domains are often seen with the largest effect sizes in psychosis (Fioravanti et al., 2012; Schaefer, Giangrande, Weinberger, & Dickinson, 2013), CHR (Fusar-Poli et al., 2012; Pukrop & Klosterkötter, 2010; Zheng et al., 2018), and MDD (Ahern & Semkovska, 2017; Goodall et al., 2018). This would suggest that these are core cognitive functions that are impacted across diagnoses (East-Richard et al., 2020), and that these domains are particularly important when screening and providing treatment for cognitive impairment at the first-episode of mental illness.

To the best of our knowledge, no previous studies have reported the prevalence of cognitive strengths in the early course of psychosis or depression. Here, we observed that up to 40.5% of the ROD group, 36.1% of the CHR group, and 16.1% of the ROP group were performing at least one s.d. higher than average in at least two cognitive tests. This would indicate that, although cognitive impairments may be pervasive in the early course of mental illness, there may also be areas of relative strength or above average performance which clinicians could harness to enhance treatment, with a focus on bolstering motivation and functioning (Allott et al., 2020).

One limitation of this study is that the CHR sample was significantly younger than the other study groups, which may have had slight impacts on the standardised z-scores calculated based on the HC group. It is possible that the prevalence of cognitive strengths and impairments in the CHR group may need to be interpreted with more caution than the other clinical groups. The study groups did also significantly differ on the other demographic variables including estimated current IQ and years of education. However, differences on these factors reflect what is commonly seen in clinical practice. The purpose of this paper was not to determine the cause of cognitive impairment (e.g. potentially due to reduced education level in clinical groups), but rather to provide clinicians with a picture of cognitive performance which they might expect to see in individuals with recent-onset mental illness. Although the individual cognitive tests may each have their own norms which are matched on these demographic variables, these are often different normative samples for each test. By creating z-scores based on the present HC sample (who were recruited from the same areas as the clinical groups), we were able to provide a standardised picture of cognitive performance across all twelve tests. It must also be acknowledged that at present, there is no universally agreed definition of a *clinically meaningful* cognitive impairment. Here, we have provided the exact prevalence of three clinical groups performing at various levels of impairment to compare to previous literature and provide clinicians with a full picture of the patterns of performance that they are likely to observe in individuals with recent-onset mental illness. We have also provided the prevalence of impairments and strengths in a HC group recruited from the same locations for comparison. This is intended as a starting point for clinical services to estimate the need for cognitive treatments. However, cognitive impairment should always be interpreted within the individual's wider clinical picture, including their premorbid functioning, and their own treatment preferences.

Given the variability in the prevalence of impairment across individual tests, this suggests that while cognitive impairment may be common even at the early stages of serious mental illness, there is likely to be high variability in the profile of cognitive performance between individuals. These findings should help us to refine our approach to cognitive interventions for individuals with early psychosis or depression. By introducing, or promoting, routine cognitive screening at service entry (Bryce & Allott, 2019; Bryce et al., 2021), those individuals who are already demonstrating severe impairment could be efficiently referred to cognitive interventions as required. Such treatment could also be tailored towards the individual's specific profile of cognitive performance. In addition to interventions which take a deficit-reduction approach, patients may also benefit from an approach which aims to highlight their cognitive strengths (Allott et al., 2020) and to work on preserving cognitive domains which are intact at the first episode to prevent further decline (Pantelis et al., 2015). Here, we used the terms ‘strengths’ and ‘impairments’, referring to the degree to which cognitive performance is above or below average, taking a normative, as opposed to individual, approach (Lezak, Howieson, & Loring, 2004). We cannot report the extent to which an individual's performance may have declined from their own previous levels (Lezak et al., 2004; Tran et al., 2021), which can be associated with poorer outcomes even when performance is still within an ‘average’ range (Raucher-Chéné et al., 2022). Assessment of an individual's cognitive performance, including their relative strengths and weaknesses, would involve a full neuropsychological assessment in which the clinician brings together the full picture of their current performance, as well as estimated premorbid performance or intraindividual assessment, and demographic variables (Lezak et al., 2004). Further, the terms ‘strength’ and ‘impairment’ may not necessarily represent how the individual perceives their own current cognition relative to previous levels, and thus, targeted treatments for cognition should be planned in consultation with the individual to assist with both their objective and subjective areas of strength and impairment. In addition, this work reports on cognitive performance at baseline only. Cognition may take a heterogeneous trajectory following the first-episode, with some individuals potentially experiencing further declines in their cognitive performance (e.g. Fett et al., 2020; Flaaten et al., 2022). Further work is required to improve our understanding of the nature and trajectory of cognition both before and following the first episode of mental illness. Such work will allow clinicians to maximise the use of time and resources, ensuring that cognition is routinely assessed and treatment tailored to the individual's particular needs based on their presentation and stage of illness.

## Conclusion

By understanding the prevalence of cognitive impairment and strength in early course mental illness, we can advocate for cognitive screening at intake, more accurately resource clinical services, and advise clinicians as to the proportion of young people who may need to access additional cognitive treatments or therapies.

## Supplementary material

For supplementary material accompanying this paper visit https://doi.org/10.1017/S0033291723001770. click here to view supplementary material
