# Supplementary material for: Prevalence of cognitive impairments and strengths in the early course of psychosis and depression
Source: Psychol Med. 2023 Jul 6;53(13):5945–57. doi: 10.1017/S0033291723001770 (PMC10520593; doi:10.1017/S0033291723001770)
Supplement: Supplementary file 1 [file S0033291723001770sup001.docx]

**Prevalence of Cognitive Impairments and Strengths in the Early Course of Psychosis and Depression**

**Supplementary Material**

***Participants***

Supplementary Table 1

*A table briefly comprising the full inclusion and exclusion criteria for the PRONIA study*

| Study Group | Inclusion Criteria | Exclusion Criteria |
| --- | --- | --- |
| All Groups | Age 15-40 | Current or past head trauma |
|  | Capacity to consent | Current or past neurological illness |
|  | Sufficient proficiency in the native language of the site | Current or lifetime alcohol dependence  IQ <70 |
| ROP | DSM-IV affective or non-affective psychotic episode within 3 months of baseline assessment | Antipsychotic medication at or above the DGPPN S3 guidelines (see Koutsouleris et al., 2018) for more than 90 days |
|  | Onset of psychotic episode within 24 months prior to baseline assessment | Onset of psychotic episode longer than 24 months prior to baseline assessment. |
| ROD | DSM-IV major depressive episode within 3 months of baseline assessment | Meeting criteria for ROP or UHR |
|  | Onset of depressive episode no longer than 24 months before baseline | Any intake of antipsychotic medication within 3 months of baseline assessment at or above DGPPN S3 guidelines |
| CHR | Meeting any of the SIPS psychosis-risk syndromes *AND/OR* meeting COGDIS criteria | Antipsychotic medication at or above DGPPN S3 guidelines for more than 30 days |
|  |  | Any intake of antipsychotic medication at or above DGPPN S3 guidelines within the month prior to baseline assessment |
|  |  | Meeting ROP criteria |

ROP= Recent-Onset Psychosis; ROD= Recent-Onset Depression; CHR= Clinical High Risk for Psychosis; DSM-IV= Diagnostic and Statistical Manual of Mental Disorders, Fourth Edition; SIPS= Structured Interview for Psychosis-risk Syndromes; COGDIS= The nine “Cognitive Disturbances” items of the Schizophrenia Proneness Instrument, Adult Version (SPI-A). DGPPN= German Association for Psychiatry, Psychotherapy, and Psychosomatics.

***Description of Cognitive Tasks***

Supplementary Table 2

*Primary outcome scores for each cognitive test.*

| **Cognitive Test** | **Cognitive Skill Measured** | **Primary Outcome Score** |
| --- | --- | --- |
| Trail Making Test, Part A  (TMT A) | Processing Speed | Total reaction time to complete the test correctly |
| Trail Making Test, Part B  (TMT B) | Mental Flexibility | Total reaction time to complete the test correctly |
| Phonetic Verbal Fluency Test (PVF) | Phonetic Verbal Fluency | Total number of correct words |
| Semantic Verbal Fluency  Test (SVF) | Semantic Verbal Fluency | Total number of correct words |
| Continuous Performance Task (CPT) | Sustained Attention | d-Prime Sensitivity Index |
| Rey Auditory Verbal Learning Test (RAVLT) | Verbal Learning & Memory | Sum of Immediate Trials 1-5 |
| Rey-Osterrieth Complex Figure (ROCF) | Visual Memory | Number of Correct Trials |
| Self-Ordered Pointing Task (SOPT) | Working Memory | Total Errors |
| Auditory Digit Span Task,  Forward (FDS) | Attention | Number of Correct Trials |
| Auditory Digit Span Task, Backward (BDS) | Auditory Verbal Working Memory | Number of Correct Trials |
| Digit Symbol Substitution Test (DSST) | Processing Speed | Total Score (Total Correct – Total Errors) |
| Diagnostic Analysis of Non-Verbal Accuracy (DANVA) | Social Cognition | Total Faces Correct |

The Trail Making Test A&B (TMT; Army Individual Test Battery, 1944) is a paper-based task in which participants are required to connect numbered circles in chronological order (TMT A) and then alternate between chronological letters and numbers (TMT B). Performance is measured in reaction times, with participants having to return to the previous circle every time an error is made. Subtracting completion time of Part A from Part B is used as an indicator of executing functioning abilities (Sanchez-Cubillo et al., 2009). Inter-rater reliability has been reported as 0.94 for Part A and 0.9 for Part B, indicating high reliability (Fals-Stewart, 1992).

Verbal Fluency was assessed using a modified version of the Controlled Oral Word Association Test from the Benton, Hamsher, and Sivan (1994) Multilingual Aphasia Examination. Two subtests assessed Phonetic Verbal Fluency (PVF; words beginning with the letter ‘F’) and Semantic Verbal Fluency (SVF; examples of real animals) in two 60-second test periods. The measure has high internal consistency of 0.83 and a test-retest reliability of 0.74 (Ruff, Light, Parker, & Levin, 1996).

Sustained Attention was assessed using the Continuous Performance Task, Independent Pairs Version (CPT; Cornblatt, Risch, Faris, Friedman, & Erlenmeyer-Kimling, 1988), in which participants are presented with three to four-digit numbers in one-second intervals and instructed to respond when two consecutive numbers are entirely identical. The main outcome measure for this test is “d-prime”, a sensitivity index used to indicate a participant’s ability to discriminate a target from distractors. Test-retest reliability for the primary outcome index was significant at 0.73 (Cornblatt et al., 1988).

The Rey Auditory Verbal Learning Test (RAVLT; Rey, 1964) was used to assess verbal learning and memory, according to the well standardised procedure described by Lezak (2004). A sum score of participants’ word list recall over five trials was used to indicate verbal learning, and recall of that same list following a 30-minute delay was used to measure verbal memory. Test-retest reliability using an alternative form has been reported as high, with coefficients ranging from 0.6-0.77 (Ryan, Geisser, Randall, & Georgemiller, 1986).

The Rey-Osterrieth Complex Figure (ROCF; Osterrieth, 1944; Rey, 1941) assessed immediate and delayed visual memory. The procedure described by Lezak (2004) was used in which participants must copy a figure to the best of their ability with no time limit. They must then draw as much as they remember immediately after the figure is removed and again following a 30-minute delay. Performance is scored based on the accuracy and placement of each element of the figure. A high inter-rater reliability of 0.91 has been reported for this measure (Delaney, Prevey, Cramer, Mattson, & Group, 1992).

In the Self-Ordered Pointing Task (SOPT; Petrides & Milner, 1982), participants must select each of a set of abstract shapes, with the shapes being rearranged following each selection. The participant must inhibit their prior selections within the set and only choose the shapes they have yet to select. The number of shapes on screen at any one time starts at four and progresses to ten. The task has a high test-retest reliability of 0.82 (Ross, Hanouskova, Giarla, Calhoun, & Tucker, 2007).

The Auditory Digit Span task (Wechsler, 1939) was used to assess both ‘Forward’ and ‘Backward’ digit span. Participants must recall a list of digits in the exact order that they were read, and then in the reverse order. In both tasks, participants start with a list of two digits which progressively increases until three consecutive errors are provided.

The Digit-Symbol Substitution Test (DSST; Copyright free version) is a component of the Wechsler Adult Intelligence Scale-Revised (Wechsler, 1981), used to assess processing speed. In this task, the participant must match a series of geometric symbols to numbers using a provided key. Final scores subtract the number of incorrect responses from the number of correct responses that the participant was able to complete in 90 seconds. Test-retest reliability of this measure is high with reported coefficients of 0.81-0.88, and the measure is thought to be particularly sensitive to cognitive impairment (Lezak, 2004).

Social cognition was assessed using the receptive facial expression subtest of the Diagnostic Analysis of Non-Verbal Accuracy (DANVA; Nowicki & Duke, 1994), in which participants had to correctly identify facial emotions (happy, sad, angry, or fearful). Both the internal consistency and the test-retest reliability of this task have been found to be acceptable, with values of 0.88 and 0.84, respectively (Nowicki & Duke, 1994).

IQ estimates were gathered using the vocabulary and matrix reasoning subscales of the Wechsler Abbreviated Scale of Intelligence (WASI; Wechsler, 2011). In the Vocabulary subscale, participants are shown a list of up to 31 words and asked to describe their meaning. Scores vary from 0-2 which indicate an incorrect answer, a fair synonym, and a good synonym, respectively. In the Matrix Reasoning subtest, participants are required to complete a pattern by selecting a component from 5 different options. Testing is stopped following three consecutive incorrect responses. For both subscales, age-scaled standard scores were computed from raw scores. The average of the two standard scores was then converted to an estimate full-scale IQ score for each participant. Both the Vocabulary and Matrix Reasoning subtests of the WASI have high inter-rater reliability, 0.94 and 0.99 respectively, and internal consistency, 0.95 and 0.94 respectively (McCrimmon & Smith, 2013).

***Additional Methods Information***

Materials are not available for this study due to agreements signed at the time of ethics approval. Analysis code could be made available. This study was not preregistered.

Supplementary Table 3

*Average raw cognitive test scores of each study group*

| **Cognitive Test** | **HC** | **ROD** | **CHR** | **ROP** |
| --- | --- | --- | --- | --- |
| TMT A | 89.03 (9.02) | 86.84 (12.54) | 85.11 (11.90) | 81.30 (15.86) |
| TMT B | 359.52 (20.46) | 353.07 (25.19) | 348.33 (31.16) | 334.50 (44.06) |
| Phonetic Verbal Fluency Test | 15.82 (5.07) | 14.09 (4.86) | 14.63 (4.80) | 12.54 (6.03) |
| Semantic Verbal Fluency Test | 25.49 (5.93) | 23.35 (6.81) | 22.77 (6.04) | 19.93 (6.31) |
| CPT d’ | 0.00 (1.89) | -0.41 (2.59) | -0.64 (2.04) | -1.69 (2.87) |
| RAVLT Trials 1-5 | 60.58 (7.51) | 58.29 (8.48) | 57.23 (8.68) | 52.05 (10.74) |
| ROCF Immediate Memory | 25.17 (5.77) | 23.85 (6.27) | 22.70 (7.02) | 19.87 (7.66) |
| SOPT Total Errors | 52.56 (5.07) | 51.29 (5.58) | 51.05 (5.53) | 47.91 (7.04) |
| Forward Digit Span | 6.46 (1.19) | 6.08 (1.25) | 6.24 (1.20) | 5.79 (1.14) |
| Backward Digit Span | 5.54 (1.40) | 5.18 (1.38) | 5.08 (1.28) | 4.53 (1.17) |
| DSST Total Score | 65.00 (11.55) | 61.00 (12.15) | 58.89 (11.90) | 49.68 (13.29) |
| DANVA Total Correct | 19.51 (2.21) | 19.30 (2.20) | 18.93 (2.35) | 18.14 (2.96) |

Notes: All values are presented as Mean (Standard Deviation). Reaction Time (TMT) and Error scores (SOPT) have been reversed so that higher values always indicate better task performance.

ROP= Recent-Onset Psychosis; ROD= Recent-Onset Depression; CHR= Clinical High Risk for Psychosis;

Supplementary Table 4

*A table showing the significant post-hoc comparisons of the proportion of each study group meeting the various levels of cognitive performance.*

|  | **1-2 SDs Above** | **No Impairment** | **1-2 SDs Below** | **> 2 SDs Below** |
| --- | --- | --- | --- | --- |
| **ROCF Immediate Memory** | HC>ROP, *p*=0.001  ROD>ROP, *p*=0.015 | HC>ROP, *p*<0.001  CHR>ROP, *p*=0.026  ROD>ROP, *p*=0.041 | ROP>HC, *p*=0.011 | CHR>HC, *p*<0.001  ROP>HC, *p*<0.001  ROP>CHR, *p*=0.038  ROP>ROD, *p*<0.001 |
| **DANVA Total Correct** | HC>ROP, *p*=0.029 | ROD>ROP, *p*=0.006 |  | ROP>HC, *p*<0.001  ROP>CHR, *p*=0.002  ROP>ROD, *p*<0.001 |
| **Forward Digit Span** | HC>ROP, *p*<0.001  CHR>ROP, *p*=0.005  ROD>ROP, *p*=0.015 |  | ROD>HC, *p*=0.045  ROP>HC, *p*=0.030 | CHR>HC, *p*=0.015  ROD>HC, *p*=0.006  ROP>HC, *p*<0.001 |
| **Backward Digit Span** | HC>CHR, *p*=0.003  HC>ROD, *p*<0.001  CHR>ROP, *p*=0.002  ROD>ROP, *p*<0.001 | HC>ROP, *p*<0.001  CHR>ROP, *p*<0.001 | CHR>HC, *p*<0.001  ROD>HC, *p*=0.002  ROP>HC, *p*<0.001  ROP>CHR, *p*<0.001  ROP>ROD, *p*<0.001 | ROP>HC, *p*=0.002  ROP>CHR, *p*=0.020  ROP>ROD, *p*=0.022 |
| **RAVLT Trials 1-5** | HC>ROP, *p*=0.006 | HC>ROP, *p*<0.001  CHR>ROP, *p*<0.001  ROD>ROP, *p*<0.001 | CHR>HC, *p*=0.033  ROP>HC, *p*<0.001  ROP>ROD, *p*=0.045 | CHR>HC, *p*=0.030  ROP>HC, *p*<0.001  ROP>CHR, *p*<0.001  ROP>ROD, *p*<0.001 |
| **TMT A** | HC>ROP, *p*=0.008 | HC>ROP, *p*=0.016 |  | CHR>HC, *p*<0.001  ROP>HC, *p*<0.001 |
| **TMT B** | HC>CHR, *p*=0.012  HC>ROP, *p*<0.001 | HC>ROP, *p*<0.001  CHR>ROP, *p*=0.007  ROD>ROP, *p*<0.001 | ROP>HC, *p*<0.001  ROP>ROD, *p*=0.002 | CHR>HC, *p*<0.001  ROD>HC, *p*=0.004  ROP>HC, *p*<0.001  ROP>ROD, *p*=0.014 |
| **Phonetic Verbal Fluency** | HC>ROP, *p*=0.002 | HC>ROP, *p*=0.048 | CHR>HC, *p*=0.007  ROD>HC, *p*=0.004  ROP>HC, *p*<0.001 | ROP>HC, *p*=0.001  ROP>CHR, *p*=0.003 |
| **Semantic Verbal Fluency** | HC>CHR, *p*=0.026  HC>ROP, *p*<0.001  ROD>ROP, *p*=0.007 | HC>ROP, *p*<0.001  CHR>ROP, *p*=0.002  ROD>ROP *p*=0.008 | CHR>HC, *p*<0.001  ROD>HC, *p*=0.020  ROP>HC, *p*<0.001  ROP>ROD, *p*=0.003 | ROD>HC, *p*=0.047  ROP>HC, *p*<0.001  ROP>CHR, *p*<0.001  ROP>ROD, *p*=0.004 |
| **SOPT Total Errors** | HC>ROP, *p*<0.001 | HC>ROP, *p*<0.001  CHR>ROP, *p*=0.002  ROD>ROP, *p*=0.032 | ROP>HC, *p*=0.006 | CHR>HC, *p*=0.019  ROP>HC, *p*<0.001  ROP>CHR, *p*<0.001  ROP>ROD, *p*<0.001 |
| **DSST Total Score** | HC>CHR, *p*=0.008  HC>ROP, *p*<0.001  CHR>ROP, *p*=0.038  ROD>ROP, *p*<0.001 | HC>CHR, *p*=0.002  HC>ROD, *p*=0.020  HC>ROP, *p*<0.001  CHR>ROP, *p*<0.001  ROD>ROP, *p*<0.001 | CHR>HC, *p*<0.001  ROD>HC, *p*=0.002  ROP>HC, *p*<0.001  ROP>ROD, *p*=0.007 | CHR>HC, *p*<0.001  ROD>HC, *p*=0.040  ROP>HC, *p*<0.001  ROP>CHR, *p*<0.001  ROP>ROD, *p*<0.001 |
| **CPT d’** | HC>ROP, *p*=0.001 | HC>ROP, *p*<0.001 | CHR>HC, *p*<0.001  ROP>HC, *p*<0.001  ROP>ROD, *p*=0.002 | ROP>HC, *p*<0.001  ROP>CHR, *p*<0.001 |
| **Composite Score** |  | HC>CHR, *p*<0.001  HC>ROD, *p*=0.006  HC>ROP, *p*<0.001  CHR>ROP, *p*<0.001  ROD>ROP, *p*<0.001 | CHR>HC, *p*<0.001  ROP>HC, *p*<0.001  ROD>HC, *p*<0.001  ROP>HC, *p*<0.001  ROP>ROD, *p*<0.001  ROP>CHR, *p*=0.004 | ROP>HC, *p*<0.001  ROP>ROD, *p*<0.001 |

Note: There were no significant differences at the ‘>2 SDs Above’ level

**Supplementary References**

Army Individual Test Battery. (1944). Army Individual Test Battery: Manual of directions and scoring. In. Washington, DC: War Department, Adjutant General's Office.

Benton, A. L., Hamsher, K. S., & Sivan, A. B. (1994). *Multilingual Aphasia Examination: Token Test*: AJA associates.

Cornblatt, B. A., Risch, N. J., Faris, G., Friedman, D., & Erlenmeyer-Kimling, L. (1988). The Continuous Performance Test, identical pairs version (CPT-IP): I. New findings about sustained attention in normal families. *Psychiatry research, 26*(2), 223-238.

Delaney, R. C., Prevey, M. L., Cramer, J., Mattson, R. H., & Group, V. E. C. S. R. (1992). Test-retest comparability and control subject data for the rey-auditory verbal learning test and rey-osterrieth/taylor complex figures. *Archives of Clinical Neuropsychology, 7*(6), 523-528.

Fals-Stewart, W. (1992). An interrater reliability study of the Trail Making Test (Parts A and B). *Perceptual and Motor Skills, 74*(1), 39-42.

Koutsouleris, N., Kambeitz-Ilankovic, L., Ruhrmann, S., Rosen, M., Ruef, A., Dwyer, D. B., . . . the PRONIA Consortium. (2018). Prediction models of functional outcomes for individuals in the clinical high-risk state for psychosis or with recent-onset depression: a multimodal, multisite machine learning analysis. *JAMA Psychiatry, 75*(11), 1156-1172.

Lezak, M. D. (2004). *Neuropsychological assessment*: Oxford University Press, USA.

Nowicki, S., & Duke, M. P. (1994). Individual Differences in the Nonverbal Communication of Affect: The Diagnostic Analysis of Nonverbal Accuracy Scale. *Journal of Nonverbal Behavior, 18*(1), 27.

Osterrieth, P. A. (1944). Le test du copie d'une figure complexe. *Archives de psychologie, 28*, 206-356.

Petrides, M., & Milner, B. (1982). Deficits on subject-ordered tasks after frontal-and temporal-lobe lesions in man. *Neuropsychologia, 20*(3), 249-262.

Rey, A. (1941). L'examen psychologique dans les cas d'encéphalopathie traumatique.(Les problems.). *Archives de psychologie*.

Rey, A. (1964). L’examen clinique en psychologie [The clinical psychological examination]. *Paris: Presses Universitaires de France*.

Ross, T. P., Hanouskova, E., Giarla, K., Calhoun, E., & Tucker, M. (2007). The reliability and validity of the self-ordered pointing task. *Archives of Clinical Neuropsychology, 22*(4), 449-458.

Ruff, R. M., Light, R. H., Parker, S. B., & Levin, H. S. (1996). Benton controlled oral word association test: Reliability and updated norms. *Archives of Clinical Neuropsychology, 11*(4), 329-338.

Ryan, J. J., Geisser, M. E., Randall, D. M., & Georgemiller, R. J. (1986). Alternate form reliability and equivalency of the Rey Auditory Verbal Learning Test. *Journal of clinical and Experimental Neuropsychology, 8*(5), 611-616.

Sanchez-Cubillo, I., Perianez, J. A., Adrover-Roig, D., Rodriguez-Sanchez, J. M., Rios-Lago, M., Tirapu, J., & Barcelo, F. (2009). Construct validity of the Trail Making Test: role of task-switching, working memory, inhibition/interference control, and visuomotor abilities. *J Int Neuropsychol Soc, 15*(3), 438-450. doi:10.1017/S1355617709090626

Wechsler, D. (1939). *The measurement and appraisal of adult intelligence* (1st ed.). Baltimore: Williams and Wilkins Corporation.

Wechsler, D. (1981). Wechsler Adult Intelligence Scale-Revised (WAIS-R) In. San Antonio, TX: Psychological Cooperation
